# 

**Published:** 1907-10-15

**Authors:** 


					﻿Dental
Register
NELVILLe S. HOFF, Editor,
Ann Arbor, Mich.
Vol. LXI-OCTOBER, 1907-- No. 10
SAMUEL A. CROCKER & CO., Publishers
OHIO DENTAL AND SURGICAL DEPOT
35 W. FIFTH STREET, CINCINNATI, 0.
PRICE, ONE DOLLAR A YEAR
Entered at the Postoffice at Cincinnati, O., as second-class mail matter
				

